# Supplementary material for: Association analysis of repetitive elements and R-loop formation across species
Source: Mob DNA. 2021 Jan 20;12:3. doi: 10.1186/s13100-021-00231-5 (PMC7818932; doi:10.1186/s13100-021-00231-5)
Supplement: Supplementary file 1 — Additional file 1 Genomic distribution of dRIP- and gRO-seq peaks. Percentages of peaks overlapping with gene, promoter2k and intergenic regions shown in the last three columns. Peaks overlapping by at least 1nt with other regions are counted according to the priority (promoter2k > gene > intergenic). Promoter2k means 2000nt upstream of a gene region. [file 13100_2021_231_MOESM1_ESM.pdf]

Additional file 1

Dataset	#Peaks	#Bases	%Gene	%Promoter2k	%Intergenic
U2OS(DRIP)	10378	9772584	85.11	6.7	8.19
U2OS(GRO)	66659	322496237	86.71	2.86	10.43
Embryo(DRIP)	7354	7060054	77.37	5.78	16.86
Embryo(GRO)	7279	19304717	91.8	5.02	3.18
S2(DRIP)	12049	9815359	75.87	5.64	18.49
S2(GRO)	5812	42665805	90.79	4.38	4.83
Seedling(DRIP)	14809	20392020	63.26	13.57	23.17
Seedling(GRO)	12485	57831988	84.85	9.63	5.52
