# Supplementary material for: Association analysis of repetitive elements and R-loop formation across species
Source: Mob DNA. 2021 Jan 20;12:3. doi: 10.1186/s13100-021-00231-5 (PMC7818932; doi:10.1186/s13100-021-00231-5)
Supplement: Supplementary file 2 — Additional file 2 Enrichment analyses of repeat classes across (A)human, (B)fruit Fly, and (C)A. thaliana. [file 13100_2021_231_MOESM2_ESM.pdf]

Additional file 2

(A)

repClass	%Genome	%Sampling	%GRO	%DRIP	log(DRIP/Genome)	Sampling(Z score)	log(DRIP/GRO)
LINE	20.96	15.67	14.56	3.30	-0.80	-23.28	-0.65
SINE	12.99	17.06	18.48	10.77	-0.08	-18.78	-0.23
LTR	8.80	7.05	5.15	1.89	-0.67	-17.64	-0.44
DNA	3.33	3.16	3.56	0.86	-0.59	-16.68	-0.62
Satellite	2.46	0.26	0.09	1.78	-0.14	17.51	1.32
Simple_repeat	1.23	1.29	1.05	1.83	0.17	10.56	0.24
Low_complexity	0.20	0.25	0.18	0.51	0.40	11.34	0.44
Retroposon	0.14	0.17	0.04	0.15	0.03	-0.39	0.54
Unknown	0.02	0.02	0.01	0.00	-1.14	-3.04	-0.90
RC	0.01	0.01	0.01	0.00	NA	-1.75	NA
snRNA	0.01	0.04	0.02	0.04	0.53	0.26	0.35
srpRNA	0.01	0.03	0.01	0.01	-0.01	-2.67	-0.20
rRNA	0.01	0.02	0.01	0.04	0.68	1.77	0.47
scRNA	0.00	0.01	0.01	0.00	-0.15	-2.91	-0.45
tRNA	0.00	0.01	0.01	0.01	0.35	1.16	-0.08
RNA	0.00	0.01	0.00	0.00	NA	-2.42	NA
Total	50.19	45.06	43.19	21.19			

(B)

		Embryo						S2					
repClass	%Genome	%Sampling	%GRO	%DRIP	log(DRIP/Genome)	Sampling(Z score)	log(DRIP/GRO)	%Sampling	%GRO	%DRIP	log(DRIP/Genome)	Sampling(Z score)	log(DRIP/GRO)
LTR	10.18	4.33	0.67	7.43	-0.14	11.86	1.05	4.35	0.97	2.54	-0.60	-8.51	0.42
LINE	4.52	2.30	0.40	4.63	0.01	12.22	1.06	2.31	0.76	2.00	-0.35	-2.25	0.42
Simple_repeat	2.62	2.66	2.46	2.00	-0.12	-10.20	-0.09	2.72	2.75	4.51	0.24	24.08	0.21
DNA	1.31	0.85	0.67	0.79	-0.22	-0.69	0.07	0.87	0.60	0.09	-1.16	-9.95	-0.82
Satellite	1.22	1.61	0.05	0.18	-0.84	-10.10	0.53	1.60	0.07	0.05	-1.41	-13.48	-0.18
RC	0.71	0.73	0.68	0.14	-0.71	-9.07	-0.70	0.77	0.69	0.09	-0.90	-11.91	-0.89
Low_complexity	0.34	0.35	0.35	0.32	-0.03	-2.00	-0.04	0.36	0.37	0.52	0.19	11.40	0.15
Unknown	0.08	0.10	0.02	0.01	-0.86	-3.75	-0.15	0.09	0.02	0.01	-1.11	-4.96	-0.58
rRNA	0.05	0.05	0.04	0.00	-1.56	-2.40	-1.45	0.05	0.04	0.00	-1.82	-3.27	-1.70
Other	0.04	0.03	0.00	0.00	NA	-2.13	NA	0.03	0.00	0.00	NA	-2.68	NA
RNA	0.01	0.00	0.00	0.00	-1.21	-0.19	-0.02	0.00	0.00	0.00	NA	-0.48	NA
ARTEFACT	0.00	0.00	0.00	0.00	NA	-0.29	NA	0.00	0.00	0.00	NA	-0.41	NA
Total	21.06	13.01	5.35	15.50				13.16	6.28	9.81			

(C)

repClass	%Genome	%Sampling	%GRO	%DRIP	log(DRIP/Genome)	Sampling(Z score)	log(DRIP/GRO)
LTR	6.70	5.08	0.43	11.99	0.25	24.90	1.45
DNA	4.42	4.56	0.62	5.86	0.12	8.47	0.98
RC	1.78	1.97	0.32	0.33	-0.73	-15.79	0.01
Simple_repeat	1.18	1.23	1.08	1.60	0.13	17.53	0.17
LINE	1.03	1.46	0.21	1.50	0.16	0.56	0.85
Satellite	0.87	0.40	0.03	0.29	-0.48	-1.71	1.06
Low_complexity	0.37	0.36	0.33	0.55	0.18	17.11	0.22
SINE	0.09	0.11	0.05	0.08	-0.07	-2.73	0.16
Other	0.05	0.05	0.01	0.04	-0.16	-1.62	0.53
rRNA	0.01	0.00	0.01	0.01	-0.09	1.97	-0.03
Total	16.51	15.22	3.08	22.25			
