# Supplementary material for: Association analysis of repetitive elements and R-loop formation across species
Source: Mob DNA. 2021 Jan 20;12:3. doi: 10.1186/s13100-021-00231-5 (PMC7818932; doi:10.1186/s13100-021-00231-5)
Supplement: Supplementary file 3 — Additional file 3 Public dRIP-seq and gRO-seq datasets used in this study. [file 13100_2021_231_MOESM3_ESM.pdf]

Additional file 3

DRIP-seq			
Species	Scource	File Name	URLs
Human	U2OS	Villarreal2020_U2OS_IP_1-1	https://sra-download.ncbi.nlm.nih.gov/traces/sra64/SRR/012121/SRR12412380
		Villarreal2020_U2OS_IP_1-2	https://sra-download.ncbi.nlm.nih.gov/traces/sra51/SRR/012121/SRR12412381
		Villarreal2020_U2OS_IP_1-3	https://sra-download.ncbi.nlm.nih.gov/traces/sra58/SRR/012121/SRR12412382
		Villarreal2020_U2OS_IP_2-1	https://sra-download.ncbi.nlm.nih.gov/traces/sra77/SRR/012121/SRR12412383
		Villarreal2020_U2OS_IP_2-2	https://sra-download.ncbi.nlm.nih.gov/traces/sra57/SRR/012121/SRR12412384
		Villarreal2020_U2OS_Input-1	https://sra-download.ncbi.nlm.nih.gov/traces/sra50/SRR/012121/SRR12412385
		Villarreal2020_U2OS_Input-2	https://sra-download.ncbi.nlm.nih.gov/traces/sra57/SRR/012121/SRR12412386
		Villarreal2020_U2OS_Input-3	https://sra-download.ncbi.nlm.nih.gov/traces/sra59/SRR/012121/SRR12412387
		Villarreal2020_U2OS_RH_1-1	https://sra-download.ncbi.nlm.nih.gov/traces/sra45/SRR/012121/SRR12412388
		Villarreal2020_U2OS_RH_1-2	https://sra-download.ncbi.nlm.nih.gov/traces/sra63/SRR/012121/SRR12412389
		Villarreal2020_U2OS_RH_2-1	https://sra-download.ncbi.nlm.nih.gov/traces/sra71/SRR/012121/SRR12412390
		Villarreal2020_U2OS_RH_2-2	https://sra-download.ncbi.nlm.nih.gov/traces/sra55/SRR/012121/SRR12412391
		Villarreal2020_U2OS_RH_2-3	https://sra-download.ncbi.nlm.nih.gov/traces/sra38/SRR/012121/SRR12412392
Fruit fly	Embryo	Alecki2020_Embryo_IP_1	ftp://ftp.ddbj.nig.ac.jp/ddbj_database/dra/sralite/ByExp/litesra/SRX/SRX544/SRX5443751/SRR8645632/SRR8645632.sra
		Alecki2020_Embryo_IP_2	ftp://ftp.ddbj.nig.ac.jp/ddbj_database/dra/sralite/ByExp/litesra/SRX/SRX544/SRX5443752/SRR8645633/SRR8645633.sra
		Alecki2020_Embryo_RH_1	ftp://ftp.ddbj.nig.ac.jp/ddbj_database/dra/sralite/ByExp/litesra/SRX/SRX544/SRX5443753/SRR8645634/SRR8645634.sra
		Alecki2020_Embryo_RH_2	ftp://ftp.ddbj.nig.ac.jp/ddbj_database/dra/sralite/ByExp/litesra/SRX/SRX544/SRX5443754/SRR8645635/SRR8645635.sra
		Alecki2020_Embryo_Input_1	ftp://ftp.ddbj.nig.ac.jp/ddbj_database/dra/sralite/ByExp/litesra/SRX/SRX544/SRX5443755/SRR8645636/SRR8645636.sra
		Alecki2020_Embryo_Input_2	ftp://ftp.ddbj.nig.ac.jp/ddbj_database/dra/sralite/ByExp/litesra/SRX/SRX544/SRX5443756/SRR8645637/SRR8645637.sra
	S2	Alecki2020_S2_IP_1	ftp://ftp.ddbj.nig.ac.jp/ddbj_database/dra/sralite/ByExp/litesra/SRX/SRX544/SRX5443763/SRR8645644/SRR8645644.sra
		Alecki2020_S2_IP_2	ftp://ftp.ddbj.nig.ac.jp/ddbj_database/dra/sralite/ByExp/litesra/SRX/SRX544/SRX5443764/SRR8645645/SRR8645645.sra
		Alecki2020_S2_RH_1	ftp://ftp.ddbj.nig.ac.jp/ddbj_database/dra/sralite/ByExp/litesra/SRX/SRX544/SRX5443765/SRR8645646/SRR8645646.sra
		Alecki2020_S2_RH_2	ftp://ftp.ddbj.nig.ac.jp/ddbj_database/dra/sralite/ByExp/litesra/SRX/SRX544/SRX5443766/SRR8645647/SRR8645647.sra
		Alecki2020_S2_Input_1	ftp://ftp.ddbj.nig.ac.jp/ddbj_database/dra/sralite/ByExp/litesra/SRX/SRX544/SRX5443767/SRR8645648/SRR8645648.sra
		Alecki2020_S2_Input_2	ftp://ftp.ddbj.nig.ac.jp/ddbj_database/dra/sralite/ByExp/litesra/SRX/SRX544/SRX5443768/SRR8645649/SRR8645649.sra
A.thaliana	seedlings	Xu2017_Seedling_IP_1	ftp://ftp.ddbj.nig.ac.jp/ddbj_database/dra/sralite/ByExp/litesra/SRX/SRX261/SRX2617507/SRR5318040/SRR5318040.sra
		Xu2017_Seedling_IP_2	ftp://ftp.ddbj.nig.ac.jp/ddbj_database/dra/sralite/ByExp/litesra/SRX/SRX261/SRX2617508/SRR5318041/SRR5318041.sra
		Xu2017_Seedling_RH_1	ftp://ftp.ddbj.nig.ac.jp/ddbj_database/dra/sralite/ByExp/litesra/SRX/SRX261/SRX2617509/SRR5318042/SRR5318042.sra
		Xu2017_Seedling_RH_2	ftp://ftp.ddbj.nig.ac.jp/ddbj_database/dra/sralite/ByExp/litesra/SRX/SRX261/SRX2617510/SRR5318043/SRR5318043.sra
		Xu2017_Seedling_Input	ftp://ftp.ddbj.nig.ac.jp/ddbj_database/dra/sralite/ByExp/litesra/SRX/SRX286/SRX2867541/SRR5626994/SRR5626994.sra

GRO-seq			
Human	U2OS	GRO_U2OS	https://www.ncbi.nlm.nih.gov/geo/query/acc.cgi?acc=GSE66928
Fruit fly	Embryo	GRO_Embryo	https://www.ncbi.nlm.nih.gov/geo/query/acc.cgi?acc=GSE41611
	S2	GRO_S2_A	https://www.ncbi.nlm.nih.gov/geo/query/acc.cgi?acc=GSE68677
		GRO_S2_B	https://www.ncbi.nlm.nih.gov/geo/query/acc.cgi?acc=GSE23543
A.thaliana	seedlings	GRO_seedlings	https://www.ncbi.nlm.nih.gov/geo/query/acc.cgi?acc=GSE117014
